# Spatio-temporal overlap between purse seine fisheries and Humboldt penguin feeding areas in northern Chile

**DOI:** 10.7717/peerj.20714

**Published:** 2026-03-17

**Authors:** Isabel Bastías-Aguilar, Thomas Mattern, Ursula Ellenberg, Maximiliano Daigre, Alejandro Simeone

**Affiliations:** 1 Programa de Magister en Recursos Naturales, Facultad de Ciencias de la Vida, Universidad Andrés Bello, Santiago de Chile, Chile; 2 The Tawaki Trust, Dunedin/Ōtepoti, Aotearoa New Zealand; 3 Global Penguin Society, Puerto Madryn, Argentina; 4 Department of Zoology, University of Otago, Dunedin/Ōtepoti, Otago, Aotearoa New Zealand; 5 Department of Marine Science, University of Otago, Dunedin/Ōtepoti, Aotearoa New Zealand; 6 Grupo de Investigación Pingüino de Humboldt (GIPH), Santiago de Chile, Chile; 7 One Health Institute, Facultad de Ciencias de la Vida, Universidad Andrés Bello, Santiago de Chile, Chile

**Keywords:** Foraging ecology, Fisheries interactions, Penguins, Spatial distribution, GPS tracking, Chile, Humboldt Current, Humboldt penguin, *Spheniscus*

## Abstract

Seabirds face increasing pressure from commercial fisheries through both direct mortality and indirect competition for shared prey resources. In Chile’s Humboldt Current System, artisanal purse-seine fisheries target small pelagic fish species such as anchoveta (*Engraulis ringens*), which also comprise key components of the Humboldt penguin’s (*Spheniscus humboldti*) diet. To investigate the potential for spatio-temporal overlap between purse-seine fisheries and penguin foraging areas, we tracked breeding Humboldt penguins from Isla Choros, Coquimbo Region, northern Chile, during the autumn and spring breeding seasons of 2022 using GPS dive loggers. We quantified penguin at-sea distribution and overlap with fishing effort data obtained from purse-seine vessels operating in the Coquimbo Region. Tracking data from 22 penguins revealed a bimodal foraging pattern linked to nest location. Penguins nesting on the eastern, mainland-facing side of Isla Choros foraged exclusively in coastal waters, while western-nesting individuals foraged both offshore and inshore, likely depending on environmental conditions. Kernel Density Estimation (KDE) analysis identified two core foraging zones: one along the mainland coast near Playa de los Choros and another southwest of the island. Purse-seine fishing effort in autumn was concentrated along the coast, overlapping substantially with the coastal foraging zone of the penguins. Nearly 60% of the penguins’ coastal core foraging area overlapped with the 50% KDE of autumn purse-seine activity. In contrast, spring fishing effort was more dispersed and located farther south, with no overlap observed between spring fishing and penguin foraging areas. These findings suggest a high likelihood of indirect resource competition between Humboldt penguins and fisheries during the autumn breeding season—a critical time when penguins are energetically constrained as central place foragers. We provide the first empirical evidence of substantial seasonal overlap between Humboldt penguins and inshore purse-seine fisheries at the Humboldt Archipelago and highlight the need to integrate seabird ecology into fisheries management. To protect vulnerable seabird populations such as the Humboldt penguin, marine spatial planning should consider critical foraging habitats and breeding schedules. Future work should aim to quantify dietary overlap, assess potential sub-lethal effects of prey depletion, and monitor the impact of small-scale, untracked fisheries within key penguin foraging areas.

## Introduction

Commercial fishing activities exert a wide range of pressures on marine ecosystems (Moore & Jennings, 2000). These include the extraction of key biological resources, reducing prey availability for native predators; physical impacts of gear such as nets, cables, and hooks on habitats and individuals; and the incidental capture of non-target species including marine mammals, birds, and turtles (Lewison et al., 2014). Together, these impacts can reshape ecological interactions, disrupt predator-prey relationships, and alter the structure and function of marine food webs (Pauly et al., 1998).

Seabirds are especially vulnerable to fisheries interactions (Votier et al., 2023). Commercial fisheries have been identified as one of the most significant global threats to seabirds, both in terms of the number of species affected and the scale of population-level impacts (Dias et al., 2019). Bycatch, or incidental mortality during fishing operations, is particularly well documented in albatrosses, petrels, shearwaters, and penguins (Tasker, 2000; Montevecchi, 2001; Trathan et al., 2015; Phillips et al., 2016; Crawford et al., 2017). Industrial longline and trawl fisheries are generally considered to pose the greatest risks in terms of severity and geographic extent (Barbraud et al., 2009), but the impacts of small-scale fisheries are increasingly recognized as potentially comparable in scope—albeit less well studied (Soykan et al., 2008; Chuenpagdee et al., 2012).

Beyond direct mortality, commercial fishing can indirectly impact seabirds through the depletion of prey resources. This type of competition, where fisheries and top predators target the same forage species, can have profound effects on seabird breeding success and population dynamics (Montevecchi, 2001). The notion of fisheries and seabirds competing for shared resources dates back over 50 years to studies of guano birds and anchovy fisheries in Peru (Schaefer, 1970). More recent work has demonstrated that such competition can lead to measurable population declines. For instance, in South Africa, overfishing of Southern African pilchard (*Sardinops sagax*) and European anchovy (*Engraulis encrasicolus*) has contributed to the ongoing decline of the African penguin (*Spheniscus demersus*), Cape gannet (*Morus capensis*), and Cape cormorant (*Phalacrocorax capensis*) (Pichegru et al., 2010; Crawford et al., 2015; Grémillet et al., 2016). In these cases, reduced prey availability near breeding colonies has directly affected seabird survival and reproductive success, with evidence that even modest no-take zones can significantly improve foraging efficiency and chick provisioning (Pichegru et al., 2009; Pichegru et al., 2010).

A similar pattern has been observed in the North Atlantic, where industrial sand eel (*Ammodytidae*) fisheries have negatively impacted kittiwakes (*Rissa tridactyla*) and other resident seabirds. Breeding success on the Isle of May declined during years of active sand eel extraction (Frederiksen et al., 2004), and broader analyses have shown correlations between sand eel catch volumes and regional seabird productivity (Cook et al., 2014; Carroll et al., 2017). These examples suggest that when prey availability drops below a critical threshold—due to either natural variability or fishing pressure—seabirds may be unable to meet their energetic demands, particularly during energetically challenging life stages such as breeding.

Despite accumulating evidence, relatively few studies have been able to robustly establish causal relationships between fisheries and seabird population trends. One key limitation is the lack of adequate time series and spatial data that capture the dynamics of fish stocks, seabird foraging behaviour, and fishing activity across relevant scales. It is particularly challenging to demonstrate that fishing effort and seabird foraging overlap both spatially and temporally during biologically critical periods (Sydeman et al., 2017; Sydeman et al., 2021). This overlap is most consequential during the breeding season, when seabirds are constrained in the distance they can travel from the nest and may be especially sensitive to localized prey depletion.

In the Humboldt Current System (HCS) of Chile, intense upwelling creates a productive marine environment that supports dense aggregations of zooplankton and pelagic fish (Thiel et al., 2007), which in turn sustain large populations of marine predators, including seabirds. The artisanal purse seine fleet operating between 29° and 33° S targets mainly anchoveta (*Engraulis ringens*), Pacific sardine (*Sardinops sagax*), Chilean jack mackerel (*Trachurus murphyi)*, and chub mackerel (*Scomber japonicus)*—species that also dominate the diets of local seabirds, including the Humboldt penguin (*Spheniscus humboldti*) (Wilson et al., 1989; Herling, Culik & Hennicke, 2005; Hernandez et al., 2020). These fisheries, while well established and economically significant, may pose an unquantified threat to penguin populations *via* direct competition for food. However, for Humboldt penguins in Chile, colony-scale overlap with purse-seine activity during breeding has not yet been quantified.

North-central Chile is home to some of the largest breeding colonies of Humboldt penguins, particularly on islands within the Humboldt Archipelago in the Atacama and Coquimbo regions (Simeone, Aguilar & Luna-Jorquera, 2018). Isla Choros (29°15′S, 71°32′W) one of the islands in the Humboldt Archipelago, lies adjacent to active fishing grounds. While previous studies have documented direct interactions between purse seine fishing and seabirds in the region—such as bycatch and vessel attraction (*e.g.*, Suazo et al., 2014; Carle et al., 2019; Simeone et al., 2021)—the extent to which penguins and fisheries compete for prey remains unclear.

We hypothesize that, because both penguins and purse seine fleets target the same forage fish species, there is a measurable spatial and temporal overlap between their respective activity areas during the penguin breeding season. In this study we aim to: (1) determine the at-sea foraging areas of Humboldt penguins breeding on Isla Choros; (2) characterize the distribution of purse seine fishing effort in adjacent waters; and (3) quantify the degree of spatial overlap between penguin foraging and fishing activity. Ultimately, we seek to contribute empirical, colony-scale baseline data to the global discussion on fisheries-seabird interactions and to inform future decisions aimed at reducing potential conflicts between conservation and the exploitation of marine resources.

## Material and Methods

### Study area & species

**Figure 1:**
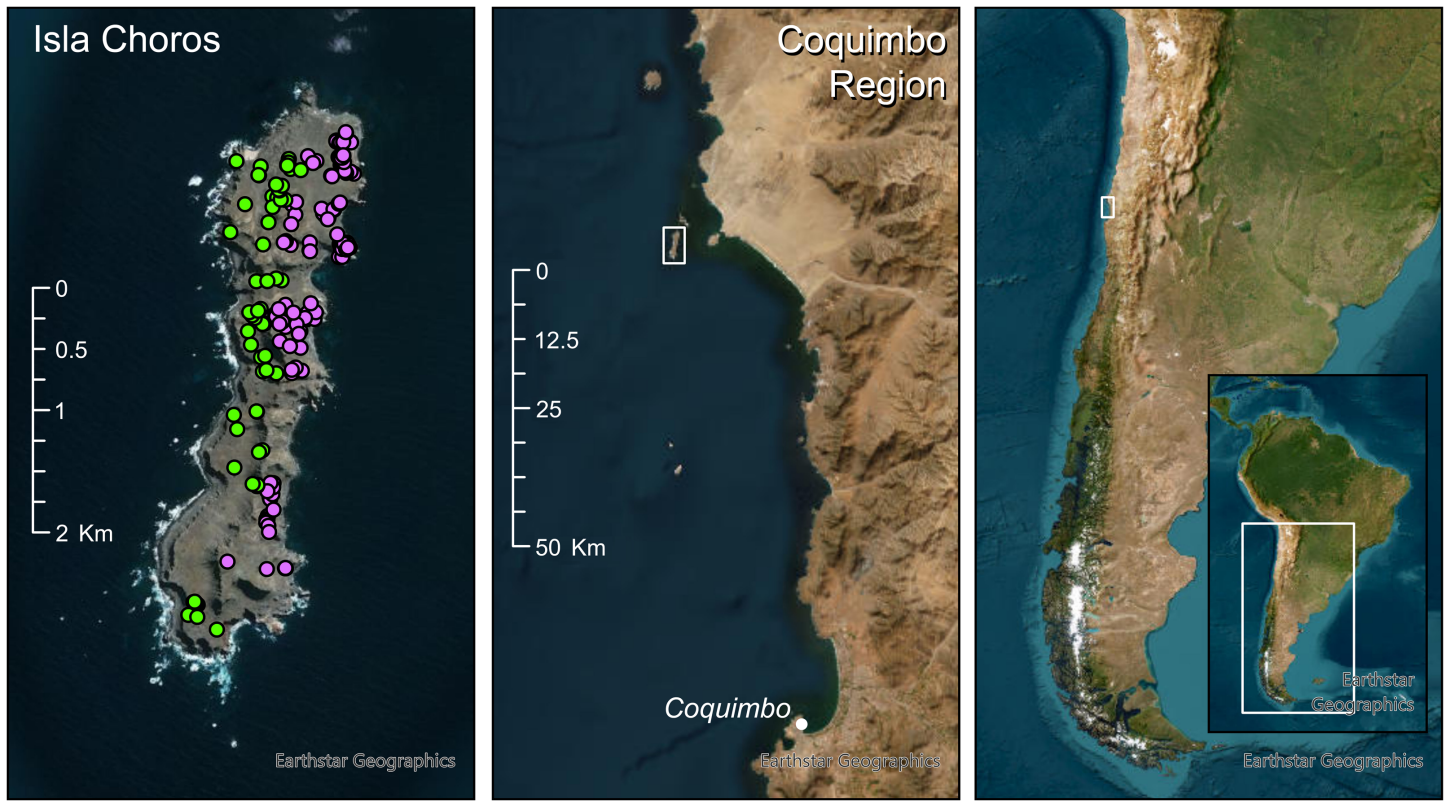
Overview of the study area and region. Left: Isla Choros, showing mapped Humboldt penguin nest sites during the 2022 autumn and spring breeding seasons. The same nest sites are active in both breeding peaks; points mark nests irrespective of season. Point colour denotes the predominant departure direction on foraging trips (green = west/ocean-facing; purple = east/mainland-facing). Centre: Location of Isla Choros within the Coquimbo Region, northern Chile. Right: Location of the study area within Chile and South America. White rectangles in the centre and right panels outline the extent of the adjacent panels to the left.

Fieldwork was conducted on Humboldt penguins (*Spheniscus humboldti*) breeding on Isla Choros (29°15′S, 71°32′W), part of the Humboldt Archipelago in the Coquimbo Region of northern Chile. The 301-hectare island lies approximately 6.5 km offshore and hosted an estimated 400 breeding pairs (Simeone, Aguilar & Luna-Jorquera, 2018) of which 202 nests were GPS mapped during the study period. The island features a central plateau that rises to an altitude of 100–120 m and runs along most of the length of the island, effectively separating the penguin breeding habitat into an eastern, mainland coast-facing, and a western, ocean facing region (Fig. 1). Of the mapped nests, 78 were located on the ocean side, the remaining 124 on the mainland coast side of the island. Breeding generally begins after moult in late austral summer and occurs in two main peaks: autumn (June–July) and spring (November–December; Paredes, Zavalaga & Boness, 2002; Simeone et al., 2002). Foraging data were collected in 2022 during the early chick-rearing (guard) phase of both breeding peaks: autumn (June) and spring (November/December).

The Humboldt penguin is endemic to the Humboldt Current System, occurring along the coasts of Peru and Chile, from approximately 5°S to 42°S (De la Puente et al., 2013). The species is classified as ‘Vulnerable’ by the IUCN Red list (IUCN, 2020) and primarily feeds on small pelagic fish, including anchoveta, Pacific sardine, Araucanian herring (*Strangomera bentincki*), and Chilean jack mackerel (Wilson & Wilson, 1989; Herling, Culik & Hennicke, 2005). The purse seine fleet operating in the Coquimbo Region focuses on anchoveta and Chilean jack mackerel (SUBPESCA, 2013; Hernandez et al., 2020). During the breeding season, Humboldt penguins typically forage within 20–35 km of their colony (Culik et al., 1998; Luna-Jorquera & Culik, 1999).

### Purse seine fleet & target species

Chilean purse-seine fisheries comprise six fleets along the coast, classified by target species, fishing area, and vessel characteristics (Vega et al., 2018). Under Chilean law, vessels ≤18 m in length are officially designated “artisanal”, and all larger vessels are designated “industrial” (Castillo & Dresdner, 2013).

The purse seine fishery in north-central Chile primarily targets anchoveta, a small, neritic pelagic fish that forms dense schools nearshore (Hernandez et al., 2020). Anchoveta exhibit short lifespans (3–4 years), rapid growth, high natural mortality, and are sensitive to environmental fluctuations (Cubillos, 2002; Bertrand et al., 2004, Bertrand et al., 2008; Castillo et al., 2021). In this region, anchoveta are mainly distributed between the coast and 20 nautical miles (∼37 km) offshore. Fishing activity is concentrated north of the port of Coquimbo, overlapping with the study area. Between 2013 and 2018, anchoveta fishing in this region was exclusively artisanal, with annual catches averaging 27,000 tonnes (Hernandez et al., 2020). In the Atacama and Coquimbo regions, a seasonal fishing ban is in effect from August to October and from December to February (Ministry of Economy, Development and Tourism, 2020).

Chilean jack mackerel, a pelagic species widely distributed throughout the Southeast Pacific, is also targeted by purse seine fleets in north-central Chile (Perea et al., 2013). In this region, it occurs primarily in two concentration zones: off Caldera (26°30′–28°S) and Coquimbo (29°30′−31°S), with a distribution extending over 70 nautical miles from shore (Hernandez et al., 2020). Historically dominated by industrial fleets, artisanal vessels have taken a growing share of catches in recent years, now accounting for more than 85% of landings in the region.

### At-sea distribution of penguins

To identify the foraging areas of chick rearing Humboldt penguins, we deployed archival AxyTrek Marine GPS loggers (TechnoSmart, Italy) on adult birds and retrieved the units by recapturing the tagged adults at the nest after 1–8 days (mean: 4.5 days); data were downloaded post-recovery. Each device included a GPS receiver, pressure and temperature sensors, and a tri-axial accelerometer, all powered by a 2,000 mAh LiPo battery. The electronics were enclosed in a streamlined epoxy housing (69 × 40 × 14 mm, 54 g); the unit’s frontal cross-sectional area and weight each represent approximately 1.5% of the penguin’s corresponding values. GPS positions were recorded at 1-minute intervals during daylight hours and at 15-minute intervals during the night. Dive depth and temperature were sampled at 1 Hz, while acceleration was recorded at 25 Hz; however, accelerometry data were not analysed for this study. AxyTrek loggers are archival tags that need to be recovered to access the data.

Devices were deployed during the early chick-rearing phase, when chicks were 1–2 weeks old, in both the autumn (15–22 June) and spring (26 November–14 December) of 2022. Adults of both sexes were selected for tagging, with sex determined from morphometric measurements (Wallace et al., 2008). In June, devices were fitted to six females and two males. Due to the state of breeding as well as accessibility of the nests, most deployments (*n* = 5) occurred in the western, ocean-facing breeding areas; only three nests from the eastern, mainland coast facing breeding areas were suitable for device deployments during the time of our visit. In November and December, eight females and five males were fitted with data loggers, eight from western and five from eastern nests. No individual was tagged more than once during the study. Pressure data were processed using custom MATLAB scripts (R2022a, The MathWorks Inc., Natick, MA, USA; available at https://github.com/ThomasMattern/matlabdiveanalysis). Dives were identified as continuous depth values ≥ 0.1 m lasting ≥ 5 s, with surface intervals defined as the periods between consecutive dives. For each dive, the last GPS position recorded during the immediately preceding surface interval was assigned as the dive location. Because GPS loggers acquired positions only when birds were at the surface, every GPS fix used in subsequent analyses represents a dive-associated location, not a transit point. GPS coverage during extended travel sequences was often limited, as surface intervals between consecutive dives were typically too brief for reliable satellite acquisition. From these dive- and GPS-linked data, the following trip-level spatial parameters were derived: (1) trip duration (time between the start of the first and end of the last dive), (2) maximum distance from the nest site, (3) total distance travelled (cumulative linear distance between dive-associated GPS fixes), and (4) mean directional bearing based on the orientation of dive-linked GPS fixes relative to the nest site.

As this represents one of the first studies to obtain simultaneous GPS and dive data from Humboldt penguins, no established behavioural thresholds exist to reliably distinguish between foraging and travelling dives based on dive parameters alone. Given the limited positional coverage during travel phases, many purely commuting dives are likely absent from the dataset, so we consider most retained dive-associated locations to represent either active foraging or prey-searching behaviour. All such locations were included in subsequent spatial analyses to describe the overall extent of at-sea activity.

We restricted spatial analyses to dive-linked GPS fixes (each location temporally matched to a verified dive event). To focus on at-sea foraging and minimise shoreline-geometry/GPS-jitter artefacts, we excluded near-shore fixes within 100 m of the Isla Choros coastline. To standardise sampling across individuals and align temporal grain with the fishery data, dive-linked fixes were aggregated to hourly centroids per bird: for each bird and hour-of-the-day, we computed the geographic mean (centroid) of all dive-linked positions in that hour. Subsequent Kernel Density Estimation (KDE) and cross-PCF analyses used these hourly centroids.

All statistical analyses were performed in R v4.2.3 (R Core Team, 2021).

To test for seasonal differences in penguin foraging behaviour, we fitted linear mixed-effects models (LMMs) for three response variables: trip duration, trip distance, and home range size. All response variables were log-transformed to improve normality, with ‘season’ as a fixed effect and ‘Bird ID’ as a random intercept to account for repeated measures. Models were fitted using the *lmer()* function from the ‘lme4’ package (Bates et al., 2015), and significance of fixed effects was assessed using the ‘lmerTest’ extension (Kuznetsova, Brockhoff & Christensen, 2017).

Home range estimates were calculated using the function *kernelUD* for KDE (Worton, 1989) in the R package ‘adehabitatHR’ (Calenge, 2024). KDE provides a smooth, two-dimensional utilisation distribution that describes the intensity of penguin space use. Bandwidth selection was performed using the selector tool *Hpi* from the Kernel Smoothing package ‘ks’ (Duong et al., 2025). KDEs were computed to derive 50%, 75%, and 95% probability contours for each season. To ensure that area calculations reflected only marine habitat, portions of the KDE polygons overlapping with the mainland were removed using the *Erase* function in ArcGIS Pro (v3.5, ESRI, Redlands, CA, USA) prior to geodesic area computation.

### Distribution of purse seine fishery

Fishing activity data were provided by the regional (Coquimbo) office of Chilean National Fisheries and Aquaculture Service (SERNAPESCA) and include artisanal purse-seine operations targeting anchoveta, Chilean jack mackerel, and chub mackerel. The area of interest for analysing fisheries-penguin overlap was defined as a 62 km buffer around Isla Choros, representing twice the maximum distance penguins were recorded traveling from the island during this study.

Artisanal purse-seine fishing in this region is carried out on a daily basis, with most vessels departing from the port of Coquimbo located ca. 110 km to the south of the study site. The data used for this study correspond to fishing sets deployed between April 2022 and February 2023 and includes, for each set, the port of departure, target species, and geographic coordinates. Per-set catch magnitudes and standardized effort metrics were not available; overlap analyses therefore used presence-only set locations. Data access was limited to artisanal vessels 12–18 m in length. Vessels under 12 metres are not legally required to carry satellite tracking devices, so no information of their fishing activities was available. Given the distance from Coquimbo, it is unlikely that a large portion of <12 m vessels would regularly operate in the vicinity of the study site. Moreover, Chilean regulations (Ministry of Economy, Development and Tourism, 2013) restrict vessels <12 m to operate within the first nautical mile of the coast. As the artisanal purse-seine fleet of the Atacama Region does not operate near Isla Choros (northern sector), only data from the Coquimbo Region were included in our analysis.

Purse seine fishing effort data were summarised as point locations of set deployments for the autumn/winter 2022 season (April–July) and the spring/summer 2022/23 season (November–February). For clarity, we refer to these as the ‘autumn’ and ‘spring’ fisheries throughout the text.

KDE was applied using the methods described above to determine spatial distribution of fishing sets to identify areas of concentrated purse-seine activity. In alignment with the seasonal reproductive cycles of penguins, the 50%, 75% and 95% probability contours were determined for the autumn and spring and periods of 2022. Geodesic area was calculated after removing portions of the resulting KDE polygons that overlapped with the mainland or fell within the 1-nautical-mile fisheries exclusion zone.

### Estimating fisheries-penguin overlap

The spatial overlap between purse-seine fishing activity (set locations) and areas used by Humboldt penguins (hourly centroids of dive-liked GPS locations, see above) during foraging trips was calculated using the *Intersect* function in ArcGIS Pro. For each corresponding KDE percentile contour, the absolute area of overlap (km^2^) was calculated, along with the relative overlap of fisheries within penguin utilisation areas (expressed as a percentage of the total penguin KDE area).

Penguin utilisation distributions were estimated from dive-associated positions pooled across seasons to reduce nest-side sampling bias. In autumn, eastern (shoreward) nests were under-represented due to fieldwork timing and nest accessibility: during our visit, few eastern nests had chicks large enough for safe handling, and many eastern nests occur under cacti (*vs.* rock shelters in the west), increasing handling risk for small chicks. An autumn-only KDE would therefore have been disproportionately influenced by western (ocean-facing) birds and shifted the colony footprint offshore. Pooling preserves the colony-level shoreward component known from eastern nests. Fishery overlap was then evaluated by intersecting this colony-level penguin KDE with season-specific purse-seine set locations (autumn: April–July 2022; spring: November 2022–February 2023). Season-specific movement statistics (*e.g.*, trip metrics, depths) were analysed separately.To assess the spatial association between penguin foraging activity and purse-seine fishing effort, we used the cross-type pair correlation function (cross-PCF) in the ‘spatstat.explore’ package in R (Baddeley & Turner, 2005; Baddeley, Rubak & Turner, 2015). The analysis was based on point pattern data derived from the hourly GPS locations of foraging Humboldt penguins and the spatial coordinates of purse-seine set locations. A marked point pattern object was constructed, and the cross-PCF was computed using both Ripley’s isotropic correction and the translation edge correction method, which account for edge effects by estimating the likelihood of observing point pairs near the boundary of the study area. The function *g*_*ij*_*(r)* describes the relative frequency of finding a type *j* point (fishing set) at distance *r* from a randomly chosen type *i* point (penguin location), compared to a spatially random distribution. Values of *g(r)*=*1* indicate spatial independence, *g(r) < 1* suggests spatial inhibition or avoidance, and *g(r) > 1* indicates clustering between the two point types at distance *r*.

### Permits and animal ethics

This study complied with all relevant national, international, and institutional guidelines for the ethical treatment of wildlife, including the Guidelines to the Use of Wild Birds in Research (Fields et al., 2023) and the ARROW guidelines for animal welfare in wildlife research. Research was carried out under permits to conduct fieldwork on Isla Choros issued by the Corporación Nacional Forestal de Chile (CONAF; N^∘^014/2021 and N^∘^019/2022). Ethics approval was granted by the Bioethics committee of the Universidad Andres Bello (N^∘^007-2023) and authorization to capture Humboldt penguins and deploy GPS devices (R. EX. No E-2021-652) was granted by the Subsecretaría de Pesca y Acuicultura de Chile (SUBPESCA).

Capture and handling were carried out by experienced personnel to minimize disturbance. Adult penguins were manually restrained for the brief period (<10 min) required to attach devices using waterproof tape. No anaesthesia or analgesia was administered, as the procedures were entirely external, minimally invasive, and did not involve tissue penetration or surgery; use of such agents was therefore not warranted. No criteria for humane euthanasia were established as no animals were injured or became moribund, and no euthanasia was performed. All birds were released at their capture site immediately after device deployment, returned to their nests, and resumed normal behaviour. No individuals were re-captured after the study concluded.

In line with the 3Rs principle:

• **Replacement**—The study addressed questions that could not be answered without working with free-ranging animals.
• **Reduction**—The number of individuals tagged was the minimum required to achieve robust statistical inference.
• **Refinement**—All capture and handling protocols were designed to minimize handling time and stress.

Procedures followed taxon-specific recommendations for the safe handling and tagging of penguins (*e.g.*, Wilson & Wilson, 1989; Hull, 1997; Ludynia et al., 2012).

## Results

### Penguin foraging behaviour

GPS tracking data were obtained from eight Humboldt penguins (six females, two males) during the autumn breeding season, resulting in 17 foraging trips. In the spring, 13 individuals (eight females, five males) were tracked, yielding 37 foraging trips. One female performed two trips that returned only a few GPS fixes at the start of each; these data were omitted. An additional short trip (<2 hrs) by another female was also excluded. The final spring dataset comprised 34 individual foraging trips.

**Figure 2:**
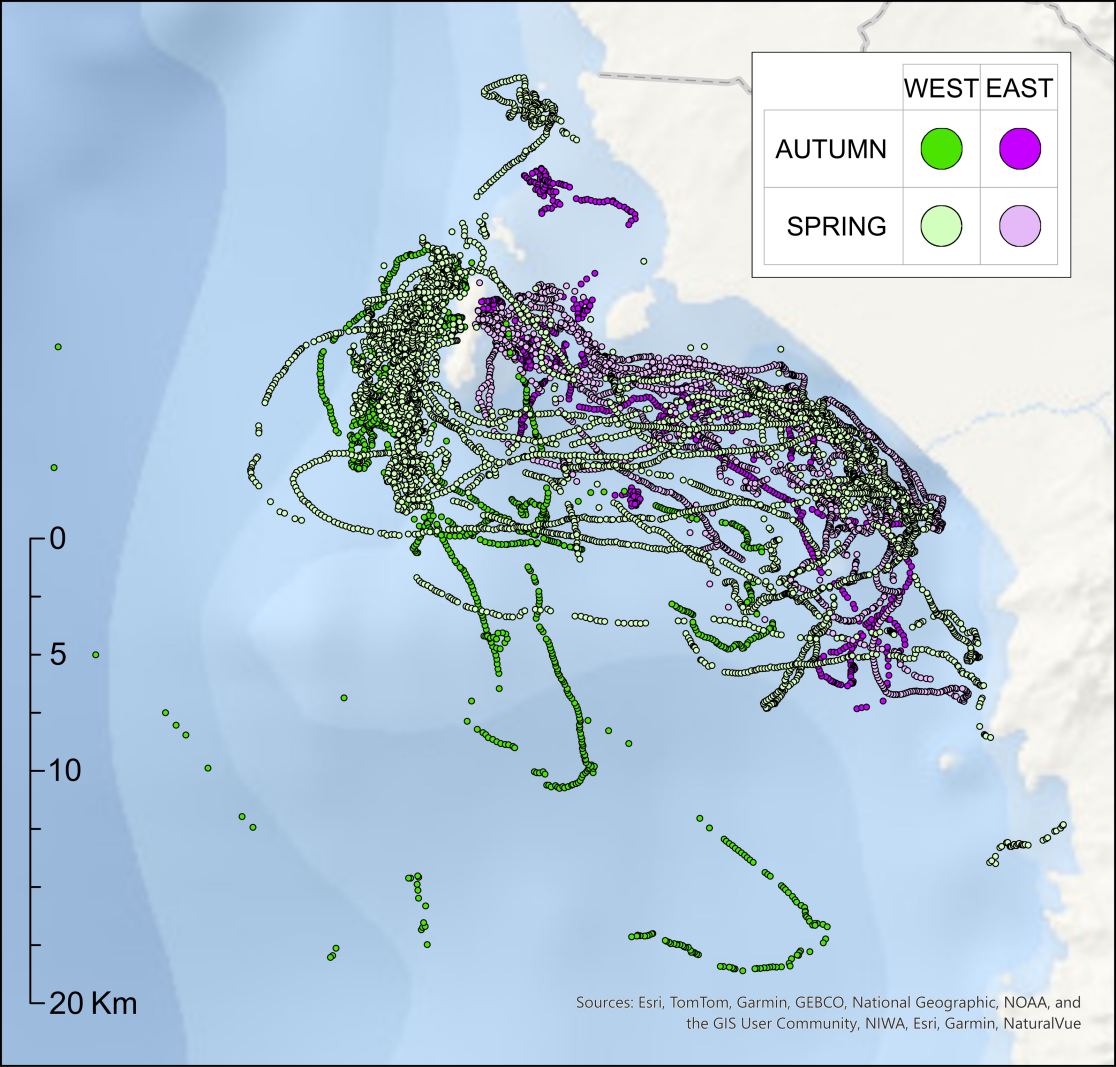
GPS locations of dive events recorded from breeding Humboldt penguins (*n* = 21) on Isla Choros during the autumn and spring breeding seasons of 2022. Each point represents a GPS fix associated with a dive event; gaps between adjacent fixes reflect either surface drifting (*e.g.*, during nighttime) or missed locations due to limited GPS reception during brief surface intervals. Marker colours correspond to the nest location of each bird (green = west/ocean-facing; purple = east/mainland-facing), with colour intensity indicating season (darker shades = autumn; lighter shades = spring).

Penguins breeding on the eastern, mainland-facing side of Isla Choros consistently foraged in nearby coastal waters. In contrast, birds nesting on the ocean-facing side exhibited greater foraging flexibility, undertaking both offshore and coastal trips (Fig. 2). This spatial divergence appears influenced by nest location: western-nesting individuals commonly exploited offshore waters up to 20 km west and southwest of the island but also foraged along the coast. Coastal foraging accounted for 18% (2 out of 11) of autumn trips and 44% (16 out of 36) of spring trips from ocean-facing nests.

**Figure 3:**
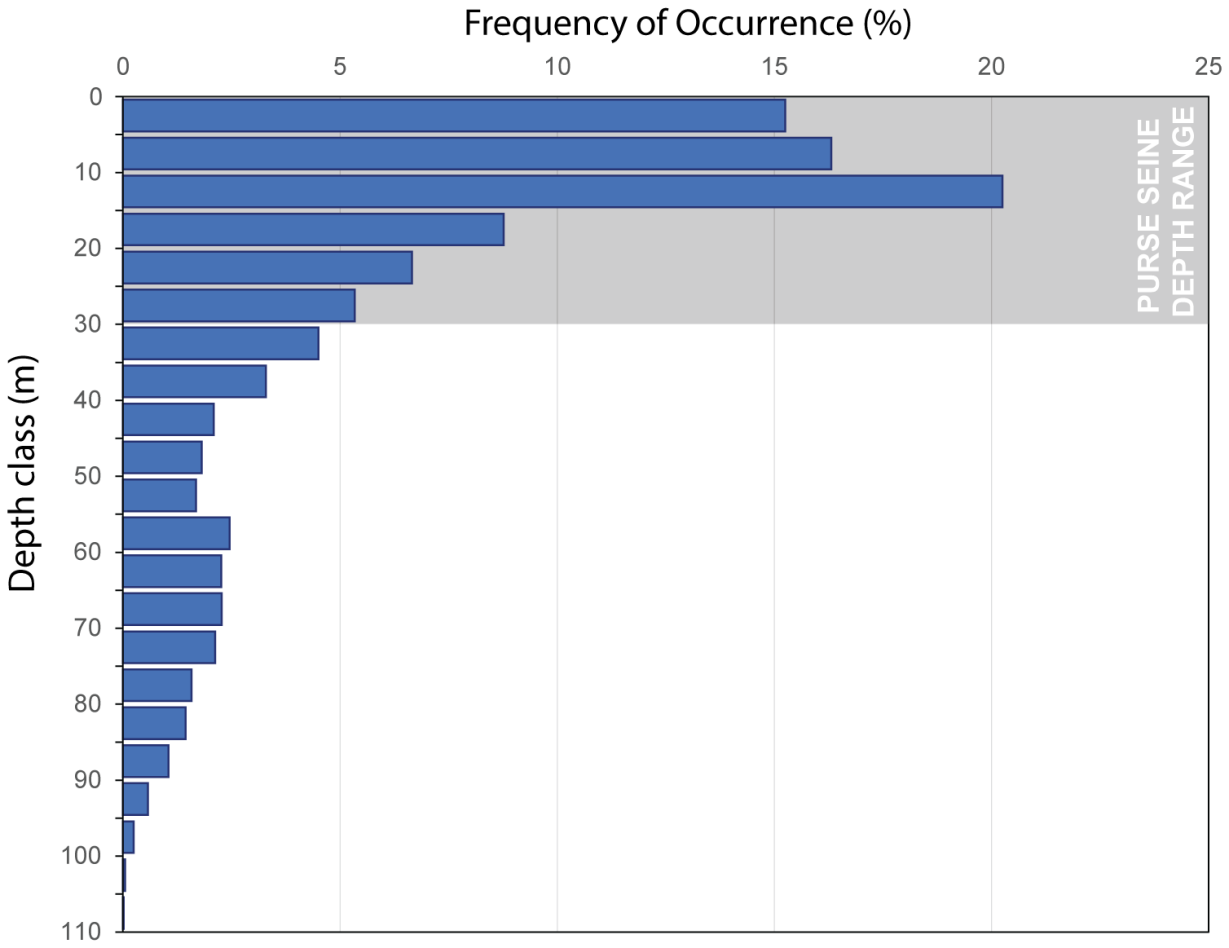
Depth-frequency distribution of Humboldt penguin dives (seasons pooled). Bars show frequency of occurrence in 5-m classes (*n* = 11,733 dives). Average purse seine depth range is indicated by grey shaded area and is based on fisheries data for the Coquimbo region summarised by Núñez Elías, Ramírez & Vásquez Pastene (2009).

A total of 28,930 dive events were recorded across both seasons (autumn: *n* = 8,559, spring: *n* = 20,371) of which 11,733 events were directly associated with a GPS fix (autumn: *n* = 3,138, spring: *n* = 8,595). Mean dive depth across all individuals ranged between 20.4 and 67.2 m (median 20.4, *n* = 16 trips) in autumn and 9.4 and 59.1 m (median: 19.0 m, *n* = 37 trips). 72.5% of all recorded dives (8,513 out of 11,733 dives) occurred in the upper 30 m of the water column (Fig. 3).

Linear mixed-effects models revealed no significant seasonal differences in core trip parameters (Table 1). Key foraging parameters were similar between autumn and spring with all 95% confidence intervals overlapping zero.

**Table 1:** Summary of linear mixed models (LMMs) evaluating seasonal differences (spring *vs.* autumn) in trip duration, trip distance, and home range of Humboldt penguins from Isla Choros, northern Chile. Fixed effects estimates are based on log-transformed response variables. Seasonal contrasts are expressed relative to the autumn/winter baseline. Median and range values are reported on the original scale. All models included bird identity (birdID) as a random intercept; singular fits indicate negligible between- individual variation in some models.

**Parameter**	**Median (range)**		**Estimate**	**95% CI**	***p***
	***Autumn***	***Spring***			
Trip duration (h)	17.2 (10.3–24.3)	15.7 (1.4–47.8)	−0.048	−0.312 to 0.217	0.724
Trip distance (km)	36.8 (16.8–68.1)	47.0 (4.3–77.2)	+0.050	−0.262 to 0.362	0.755
Home range (km^2^)	9.7 (5.4–27.2)	16.0 (1.7–29.5)	−0.008	−0.413 to 0.397	0.970
Mean depth (m)	20.4 (10.4–67.2)	19.0 (9.4–59.1)	−0.013	−0.430 to 0.404	0.952
Max depth (m)	54.0 (20.5–110.6)	66.1 (22.5–100.2)	−0.172	−0.110 to 0.454	0.245

### Spatial use and KDE

**Figure 4:**
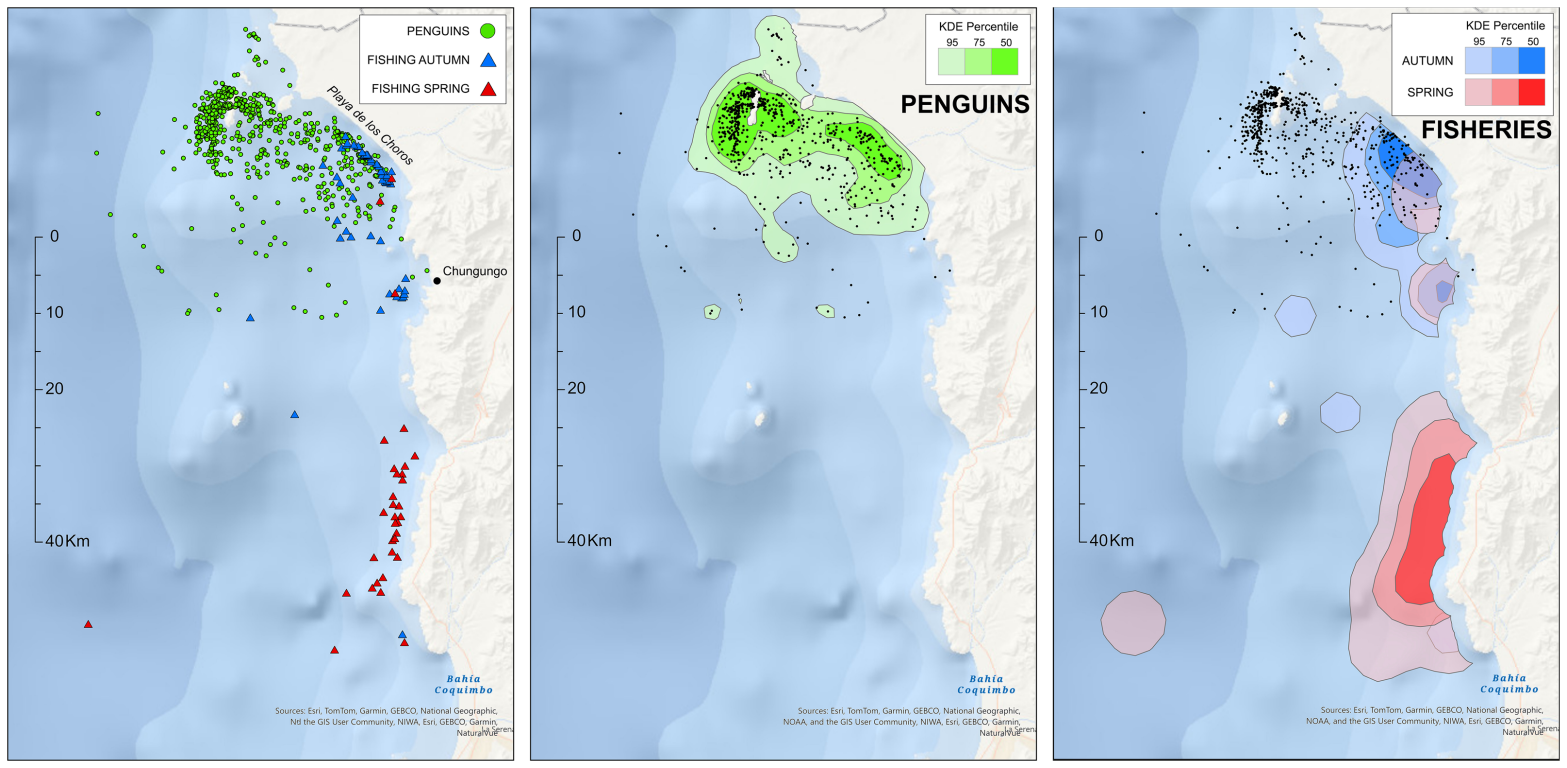
Kernel Density Estimation (KDEs) of Humboldt penguin foraging locations and purse-seine fishing activity around Isla Choros, northern Chile. The left panel shows the raw spatial data used to generate the KDEs: green dots represent hourly averaged penguin GPS positions, while blue and red triangles indicate purse-seine fishing sets during the autumn and spring seasons, respectively. The centre panel displays 95%, 75%, and 50% utilisation distributions for breeding Humboldt penguins. The right panel shows equivalent utilisation areas for purse-seine fishing effort, with blue indicating autumn (April–June 2022) and red indicating spring (November 2022–February 2023). Black points in the centre and right panels replicate penguin GPS locations for visual reference.

Due to limited autumn deployments on the eastern side, data from both seasons were pooled to improve spatial coverage (Fig. 4). While this helped balance coastal and offshore foraging data, a residual bias toward ocean-facing nests remained. Of the 30,045 pooled GPS fixes (autumn: 5,725; spring: 24,320), 736 remained after hourly averaging. KDE analysis revealed a bimodal distribution of foraging effort, reflecting nest-side differences.

Two distinct 50% KDE areas were identified (Fig. 4): one located west/southwest (and partly east) of Isla Choros, and another along the ∼14 km stretch of Playa de los Choros on the mainland coast. Combined, the core foraging areas (50% KDE) covered 77.6 km^2^, while the broader 95% KDE area extended over 380 km^2^ (Table 2).

**Table 2:** KDE areas of chick-rearing Humboldt penguins from Isla Choros, northern Chile, and purse seine fisheries operating out of Port Coquimbo in the austral autumn (April–July 2022) and spring (November 2022–February 2023). Note that penguin data from both seasons were pooled due to statistical similarity and to increase sample size of KDE. Seasonal differentiation of purse seine fishery was maintained due to significant differences in distribution of fishing efforts.

	**Number of locations (n)**	**Calculated KDE area (km**^2^)		
		***95%***	***75%***	***50%***
Humboldt penguins	736	379.3	172.5	77.7
Purse-seine autumn	54	218.3	90.8	32.6
Purse-seine spring	43	392.0	137.5	62.1

### Purse-seine fishing activity

Fisheries data for the study period included 54 purse-seine sets in autumn (April–July 2022) and 43 in spring (November 2022–February 2023). Autumn fishing was concentrated inshore, east and southeast of Isla Choros, remaining close to the 1-nautical-mile limit. All 54 autumn sets targeted anchoveta. The spatial distribution showed two main areas of effort: one along Playa de los Choros (42 sets), overlapping penguin foraging areas, and another smaller cluster (9 sets) 2–5 km offshore from Chungungo (Fig. 4). Only two sets occurred more than 15 km from the coast. The 50% KDE area of autumn fishing activity covered 32.5 km^2^ (Table 2).

In contrast, spring fishing effort was concentrated further south approximately 20–50 km north of Port Coquimbo, and ca 30 km south of the penguin’s core inshore foraging areas. Spring sets primarily targeted Chilean jack mackerel (26 sets) and chub mackerel (10 sets), with just 5 sets for anchoveta. Within the penguins’ foraging range, only 11 spring fishing sets occurred—three for anchoveta, and eight for Chilean jack mackerel. Spring fishing activities covered substantially larger core areas, with the 50% KDE spanning 483.6 km^2^—a nearly 15-fold increase compared to the autumn fishery.

### Overlap between penguins and fisheries

Spatial overlap between penguin foraging areas and purse-seine fisheries was notably higher in the autumn season than in spring. Overlap estimates based on 50% KDE polygons are shown in Table 3. Overall, 19.1% of the penguins’ 50% core KDE area overlapped with the fishery’s 50% KDE area during autumn. When focusing specifically on the penguins’ core foraging area along the mainland coast (excluding the western regions), this overlap rose to 56.7%. In contrast, there was no overlap between the 50% KDE areas of penguins and the fishing fleet in spring. This marked seasonal difference is further supported by the cross-pair correlation function (cross-PCF) analysis.

**Table 3:** Overlap of KDE areas of purse seine fisheries operating out of Port Coquimbo in the austral autumn and spring 2022 with foraging areas of chick-rearing Humboldt penguins from Isla Choros, northern Chile. Overlap is expressed as area in km^2^; relative overlap of fisheries in relation to penguin KDE areas (%) is provided in parentheses. KDEs are visualised in Fig. 4. Penguin KDEs are pooled across autumn and spring breeding to reduce deployment-location bias toward the western sector; see Methods for rationale and details of the pooling procedure.

**Penguin KDE**	**Absolute and relative KDE overlap**	
	**Autumn fisheries**	**Spring fisheries**
**50% area**	14.8 km^2^ (19.1%)^*^	no overlap (0%)
**75% area**	41.3 km^2^ (23.9%)	no overlap (0%)
**95% area**	108.4 km^2^ (28.6%)	38.9 km^2^ (10.3%)

**Notes.**

* The penguins’ 50% area close to the mainland coast along Playa de los Choros (see Fig. 4) comprises 26.2 km^2^ resulting in a 56.7% overlap of the autumn fisheries’ 50% area.

**Figure 5:**
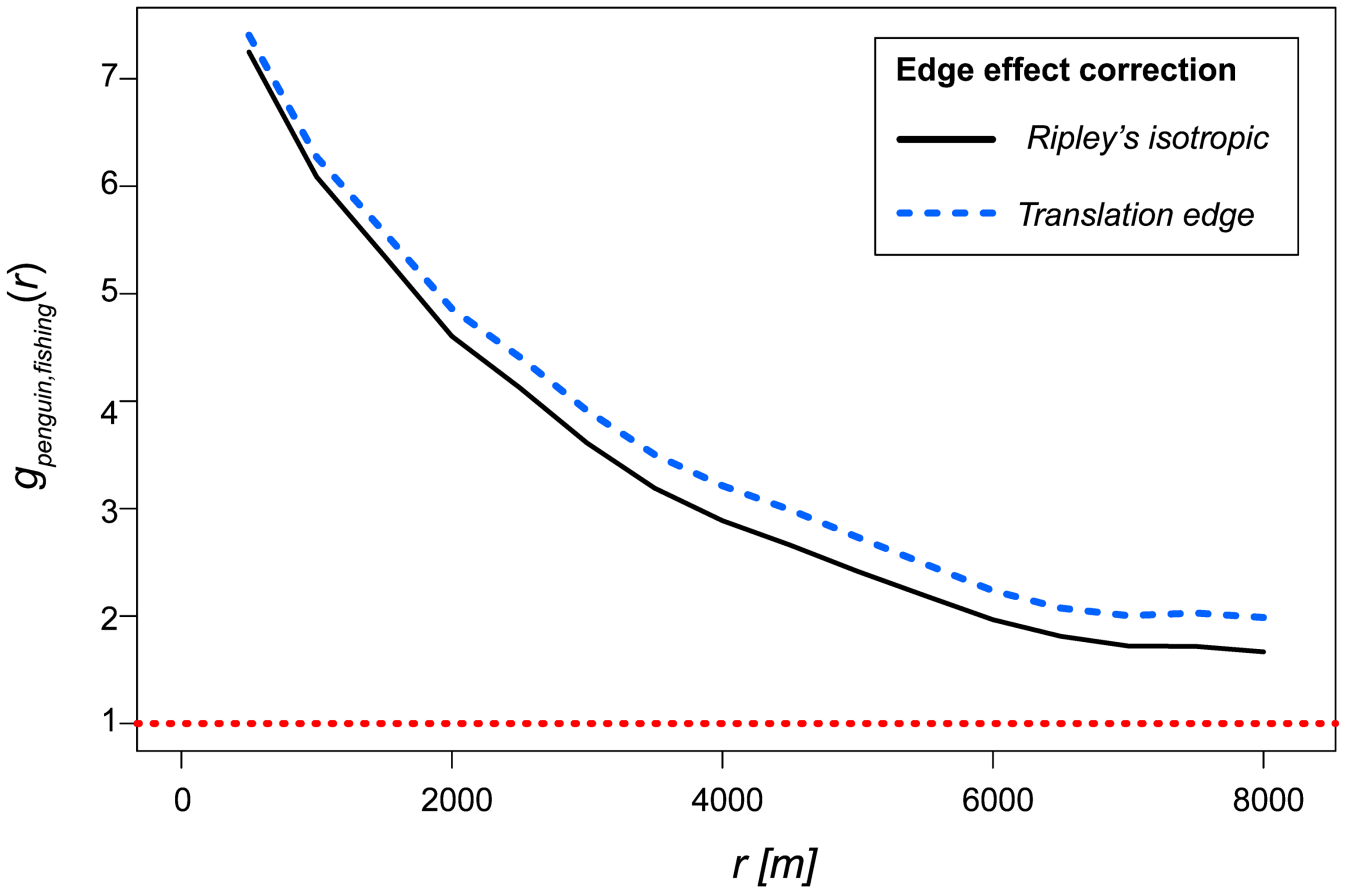
Cross-type pair correlation function (cross-PCF) showing the spatial association between penguin foraging locations and autumn purse-seine fishing activity around Isla Choros, plotted as a function of distance *r*(*m*). Values of *g*(*r*) > 1 indicate a higher-than-random likelihood of co-occurrence at distance r, suggesting spatial clustering. The analysis was performed using two edge correction methods: Ripley’s isotropic (solid black line) and translation edge correction (dashed blue line). Both indicate strong clustering at short distances, with a peak around 700 m and persistent association over several kilometres. The red dotted line denotes the null expectation under spatial independence *g*(*r*) = 1. Note: similar analysis using spring fishing data produced no spatial correlation (*i.e., g*(*r*) ≈ 1).

Cross-PCF analysis revealed a strong spatial association between penguin foraging locations and autumn fishing activity. The correlation function peaked at *g(r)* = 7 around 700 m, indicating a seven-fold higher likelihood of penguin-fishing co-occurrence at this distance than expected under spatial randomness (Fig. 5). This clustering effect declined gradually but remained elevated (*g(r)* ≈ 2 at 8 km), suggesting sustained spatial association over several kilometres. Both isotropic and translation edge corrections produced similar results, with slightly higher *g(r)* values from the translation correction. In contrast, during spring, *g(r)* ≈ 1 across all spatial scales, indicating no detectable spatial association between penguin foraging and fishing activity

## Discussion

Our study revealed consistent foraging patterns in Humboldt penguins that were closely linked to the longitudinal distribution of their nests on Isla Choros. Penguins breeding with access to the island’s eastern shores foraged exclusively towards the mainland coast, particularly off Playa de los Choros. In contrast, individuals departing from the west coast either foraged offshore to the south and southwest or, in some cases, used the same coastal foraging grounds as the eastern birds. The consistency of these patterns underscores the importance of the mainland coastal zone as a key foraging habitat. However, this also brings the penguins into direct spatial overlap with purse-seine fisheries targeting anchoveta during the autumn fishing season. While environmental factors (*e.g.*, SST, chlorophyll-a concentration) likely structure prey fields, fine-scale products for our coastal domain were not available, and our analysis was designed to quantify fishery overlap rather than to model habitat.

## Bi-modal foraging distribution

Isla Choros forms a substantial topographical barrier that shapes the foraging distribution of Humboldt penguins breeding on its eastern and western shores. The island’s elevated central plateau effectively prevents penguins nesting on one side from accessing the coast on the other. Similar effects of physical separation have been documented in other seabird species.

For instance, on the Falkland Islands/Islas Malvinas, the foraging distribution of Southern Rockhopper penguins (*Eudyptes chrysocome*) differs markedly depending on whether the birds breed on the northeastern or western coastlines, with little to no overlap in their at-sea ranges (Masello et al., 2010). Interestingly, similar but less clearly defined patterns were also observed in Magellanic penguins (*Spheniscus magellanicus*) breeding on the same island. While individuals from an east-facing colony foraged exclusively to the east, those from a southern colony primarily foraged southwest, with some individuals also reaching the eastern foraging grounds. These patterns are broadly consistent with those observed in Humboldt penguins on Isla Choros.

One commonly cited explanation for such spatial segregation is the reduction of intra-specific competition (Grémillet et al., 2004, p. *e.g.*; Masello et al., 2010; Corman et al., 2016). However, in the case of Isla Choros, this seems unlikely, as penguins from the western side also foraged extensively in the same coastal areas used exclusively by eastern breeders. Instead, the observed divergence in foraging behaviour is more plausibly explained by proximity and ease of access to spatially distinct foraging hotspots. This mechanism has been proposed to underlie at-sea distribution patterns in Balearic shearwaters (*Puffinus mauretanicus*) (Louzao et al., 2011) and Gentoo penguins (*Pygoscelis papua*) (Masello et al., 2017). Similarly, tawaki/Fiordland penguins (*Eudyptes pachyrhynchus*) have been shown to adopt a straightforward foraging strategy, swimming along a set trajectory until suitable feeding conditions are encountered (Otis et al., 2025). Such a strategy may also apply to Humboldt penguins breeding on Isla Choros’ western shores—either foraging to the southwest or continuing around the island towards the coast when prey availability is poor at the initially targeted foraging hotspot.

This would suggest that the coastal region along Playa de los Choros offers more stable and predictable foraging conditions. This in turn may help explain the higher concentration of nests on the island’s eastern side. However, other factors likely contribute as well, including the availability of suitable nesting habitat (*e.g.*, Colombelli-Négrel & Iasiello, 2023) and differing levels of human disturbance (Ellenberg et al., 2006). Isla Choros’ western coast, for example, is frequently visited by local seaweed collectors, which may influence nest site selection. Regardless, the consistent use of the mainland coast by all tracked penguins highlights the critical importance of this foraging area. The fact that this same region is heavily exploited by the autumn anchoveta fishery may also provide insights into the prey species that penguins are targeting.

### Importance of the mainland coast foraging grounds

Humboldt penguins in northern Chile are known to feed primarily on schooling fish, particularly anchoveta. Given the substantial spatial overlap with the autumn fishery, it is reasonable to assume that both penguins and the fishing fleet target the same species near the mainland coast. Prey appears to be consistently abundant along the shores of Playa de los Choros year-round, as evidenced by eastern breeders exclusively foraging inshore during both the autumn and spring breeding seasons.

Western breeders, in contrast, appear to rely on these inshore grounds as a fallback when offshore areas southwest of the island fail to provide adequate foraging conditions. These suboptimal conditions may relate to either prey abundance or prey quality. One possible explanation for the higher prey abundance along the coast lies in the oceanographic characteristics of the area. Zooplankton—key prey for pelagic fish species—tend to concentrate at shallower depths due to bathymetric constraints; as depth increases, prey availability decreases exponentially, thus, fish are generally more successful foraging over shallower seabed (Aarflot, Dalpadado & Fiksen, 2020). Furthermore, the presence of the coastline itself may act as a physical barrier that aggregates fish, making coastal foraging grounds more predictable (*e.g.*, Abookire, Piatt & Robards, 2000; Hunter-Thomson, 2011). This predictability benefits penguins and attracts the autumn purse-seine fleet targeting anchoveta.

### Fisheries and penguins targeting same resource

During the autumn season, inshore fisheries in the Coquimbo region focus almost exclusively on anchoveta, with purse seine fishing activity concentrated just outside the 1-nautical-mile limit off Playa de los Choros (Fig. 3). This region overlaps substantially with the inshore foraging grounds of Humboldt penguins from Isla Choros. Nearly two-thirds (56.7%) of the penguins’ core foraging area—defined by the 50% KDE—also falls within the core fishing zone for the autumn anchoveta fleet (Table 3).

Moreover, fishing activity in autumn is much more spatially concentrated compared to spring, when fishing grounds are an order of magnitude larger and target fish species of lesser importance to penguins. Set-level target labels indicate that, during our spring window, most purse-seine deployments were recorded as jack mackerel (listed as “Caballa” or “Jurel”, *n* = 34 sets), with anchoveta present but less frequent (*n* = 5 sets). This greater spatial overlap during autumn greatly increases the potential for interactions between penguins and the fishery—either indirectly through resource competition, or directly *via* bycatch.

The likelihood of resource competition is high. While we lack quantitative diet data from Humboldt penguins on Isla Choros, video logger footage strongly suggests that the birds are targeting schooling fish such as anchoveta and sardine (Ellenberg et al., unpubl. data; https://youtu.be/I42_HIp5SjQ), consistent with diets reported for populations further north and south (Herling, Culik & Hennicke, 2005; Hernandez et al., 2020). Although the degree of impact is difficult to quantify, there are several indications that competition may be affecting the penguins.

First, Humboldt penguins in this region exhibit two distinct breeding peaks per year: one in autumn (May) and another in spring (October) (De la Puente et al., 2013). Nest numbers and breeding success tend to be lower in autumn than in spring, a pattern that has been linked to both prey availability and rainfall (Simeone et al., 2002). Given that rainfall is scarce in the semiarid Coquimbo region, foraging conditions are likely a key limiting factor. The peak anchoveta fishing season coincides with the autumn breeding period, indicating high commercial availability of this prey. This overlap implies that reduced or unreliable prey availability, potentially due to direct competition with the purse-seine fleet, could contribute to lower reproductive output in the autumn. Beyond spatial co-use, changes in fish biomass and schooling behaviour driven by harvesting can also influence prey detectability and predator foraging success, with potential knock-on effects on reproductive output (*e.g.*, fewer schools at lower biomass; fisheries-induced shifts in shoaling/schooling). While such mechanisms are plausible in our system, our data do not include school morphology or catch-per-unit-effort, so we treat them as hypotheses for future work.

Second, our tracking data show a notable shift in foraging behaviour among western breeders during the autumn season. While the fishing fleet was active near the island, only 2 out of 11 foraging trips by western breeders were directed toward the coast. In contrast, during spring, nearly half of the foraging trips from the same group of birds utilized the inshore zone.

Of course, these interpretations remain uncertain in the absence of quantitative dietary or prey availability data. Nevertheless, highlighting potential resource competition between Humboldt penguins and commercial fisheries is important. Demonstrating competition between seabirds and fisheries is notoriously challenging, and few studies have conclusively shown that fisheries reduce prey availability for seabirds (Sydeman et al., 2017). The situation is further complicated by fluctuations in anchoveta stocks, which have alternated between “underexploited” and “overexploited” classifications over the past decade (SUBPESCA, 2015, SUBPESCA, 2022).

### Overlap between penguins and purse seine fisheries in autumn

There was substantial spatial overlap between the autumn purse-seine fishery and the core foraging grounds of Humboldt penguins. While offshore foraging areas southwest of Isla Choros did not overlap with purse-seine fishing activities, nearly 60% of the penguins’ foraging range along the mainland coast overlapped with the autumn purse-seine fleet (Table 3, Fig. 3). This high overlap is largely explained by the fact that during this time of the year (March throughout July) the local purse-seine fishery is fully exploiting anchoveta in the region (Ministry of Economy, Development and Tourism, 2020).

Seabirds are most spatially constrained during the breeding season, when they behave as “central place foragers”—returning frequently to the colony to provision chicks or relieve their mates (Boyd et al., 2015; Boyd et al., 2017). For penguins, these constraints are even more pronounced due to their flightlessness, which limits their range and foraging speed. At the same time, energy demands are highest during chick-rearing, increasing the risk that depleted prey supplies, through natural variability or competition with fisheries, will have adverse effects (Sydeman et al., 2017). Competition during such periods can reduce adult body condition and chick provisioning, with direct implications for population dynamics, including adult survival and juvenile recruitment (Sherley et al., 2015; Sherley et al., 2017). Based on our data, the purse-seine fishery appears to co-utilize, and likely affect, key prey resources during the penguins’ autumn breeding season—a critical phase for population viability.

Another factor to consider is how overlap with fisheries may vary depending on the breeding phase. Chiu Werner et al. (2011) found that Humboldt penguins from Punta San Juan travelled an average maximum distance of 11.6 km (range: 5.3–20.6 km) during chick-rearing, but up to 68.5 km (range: 64.9–71.9 km) during incubation. Our study focuses exclusively on the chick-rearing phase, when movements are more constrained. It is therefore reasonable to assume that penguins range farther during incubation, potentially increasing their overlap with purse-seine operations if they extend their coastal foraging areas southward.

Seasonality also plays a role in shaping foraging behaviour and should be considered in marine spatial planning and habitat protection (Chiu Werner et al., 2011). Although we did not detect significant seasonal differences in foraging distance, other studies have reported that breeding Humboldt penguins can forage up to 90 km from their colony (Culik & Luna-Jorquera, 1997). However, during the chick-rearing phase, penguins are typically found within 35 km of the colony (Culik & Luna-Jorquera, 1997; Culik et al., 1998; Boersma et al., 2007). Our data align with these findings: penguins in our study showed a median maximum distance from the colony of 17.1 km in spring and 11.9 km in autumn.

Future studies should explore how the presence of active fishing fleets may alter penguin foraging behaviour, particularly whether fisheries pressure affects trip distance, duration, or spatial use during different breeding phases. Such information would be invaluable for assessing potential sub-lethal effects of resource competition and guiding effective fisheries management.

### Potential effects of resource competition

Cury et al. (2011) demonstrated how seabird reproductive success is closely tied to prey availability. Their findings show that breeding success increases with prey abundance and plateaus once food becomes plentiful. However, it begins to decline when prey abundance drops below one-third of maximum levels. If the local purse-seine fishery reduces anchoveta stocks accessible to Humboldt penguins during the autumn breeding season, a decline in breeding success is a likely consequence. Determining the threshold of resource extraction that negatively impacts penguin foraging success is therefore a critical research question—essential for informing marine spatial planning and setting sustainable fishing quotas.

This is particularly important for the conservation of vulnerable species such as the Humboldt penguin, whose reproductive success depends directly on food availability in waters adjacent to their breeding colonies.

The fishing effort data used in this study overlapped—both spatially and temporally—with the penguins’ foraging trips during both breeding seasons in 2022, offering a realistic snapshot of the extent of resource co-use. According to Hernandez et al. (2020), the general area targeted by the purse-seine fleet has remained consistent over time in the study area, even though the exact positions of fishing sets may shift year to year. For this study, we analysed vessels between 12 and 18 m in length, which are required to carry satellite positioning systems. However, vessels under 12 m are exempt from this requirement, and their activity is not recorded. It is highly likely that these smaller boats, although in a much smaller magnitude, also extract prey resources critical to penguins, particularly during sensitive breeding periods.

Between July and October, no fishing trips were recorded in the study area, reflecting the period when anchoveta extraction is banned (Ministry of Economy, Development and Tourism, 2020). Changes in fishing activity within the penguins’ foraging range are likely to influence their behaviour. For example, Bertrand et al. (2012) reported that changes in fishery presence were associated with increased foraging trip distances and durations in seabirds.

### Bycatch risk in inshore purse seine fisheries

Finally, fisheries can affect penguins not only through resource competition but also *via* incidental mortality. Artisanal gillnet fisheries, widespread along the Chilean coast, pose a serious threat through entanglement (Simeone, Bernal & Meza, 1999; Pütz et al., 2011; Suazo et al., 2014; Crawford et al., 2017). These fisheries are typically conducted by vessels under 12 m in length, which are not required to carry tracking systems or report set locations. As a result, assessing the true impact of gillnet fisheries on penguin populations remains difficult. While penguin entanglement risk is highest in gillnets, purse-seine interactions are reported but appear comparatively infrequent (Crawford et al., 2017).

Our dive records indicate that penguins concentrate activity in the upper water column: 72.6% of dives were ≤30 m and 84.3% were ≤50 m (Fig. 3). Inshore purse-seine sets for small pelagics in the region operate predominantly in the upper 30 metres (Núñez Elías, Ramírez & Vásquez Pastene, 2009), so penguins and purse-seiners can exploit the same near-surface layer at the same time. This supports two points: (i) vertical co-use consistent with potential resource competition, and (ii) a non-zero risk of purse-seine bycatch where sets and foraging coincide. The comparatively lower number of reported purse-seine incidents suggests penguins are more likely to avoid or escape purse-seine gear than gillnets, though reporting differences may also contribute (Crawford et al., 2017).

## Conclusions

Seabird populations are declining globally (Paleczny et al., 2015; Dias et al., 2019), with overexploitation of forage fish among the key drivers (Pichegru et al., 2010; Bertrand et al., 2012). Humboldt penguins have experienced notable population declines in recent decades. While the causes are multifaceted, heavy commercial exploitation of their main prey species has been identified as a primary factor (IUCN, 2020). For example, the Spanish sardine, a documented prey item in the penguin’s diet (Herling, Culik & Hennicke, 2005), has reached a state of depletion or collapse, with catch volumes in the past two decades falling well below historical levels (SUBPESCA, 2022).

Although anchoveta stocks are currently classified as underexploited in the Atacama and Coquimbo regions (SUBPESCA, 2022), past fluctuations suggest that overexploitation may recur, as seen in earlier years (SUBPESCA, 2015). Historical evidence from the onset of large-scale industrial anchoveta and Araucanian herring fisheries along the Peruvian coast in the 1960s indicates that prey depletion has likely limited the capacity of seabird populations, such as Humboldt penguins, to recover to historical population levels (Jahncke, Checkley & Hunt, 2004).

Our findings highlight the urgent need for integrated marine spatial planning that accounts for the ecological requirements of vulnerable top predators such as the Humboldt penguin. The substantial spatial overlap between penguin foraging grounds and commercial fisheries, particularly during energetically demanding breeding periods, underscores the potential for both direct and indirect impacts on reproductive success and population viability. To ensure the long-term sustainability of both penguin populations and fisheries, future efforts should focus on establishing thresholds for resource extraction, improving monitoring of artisanal fishing activities particularly on vessels <12 m, and promoting adaptive management strategies that minimize competition and bycatch. Protecting critical foraging habitats—especially in regions like the Humboldt Archipelago where penguins and fisheries converge—will be essential for balancing conservation priorities with socio-economic interests along the Chilean coast.

## Supplemental Information

10.7717/peerj.20714/supp-1 Supplemental Information 1 ARRIVE checklist

## Additional Information and Declarations

### Field Study Permissions

The following information was supplied relating to field study approvals (i.e., approving body and any reference numbers): Permits to conduct research on Isla Choros were issued by the Corporación Nacional Forestal de Chile (CONAF).

### Data Availability

The following information was supplied regarding data availability: The satellite data are available at the Movebank Repository, Study ID: 5371230531.
https://doi.org/10.5441/001/1.725
